# Efficacy and Safety of Serratus Anterior Plane Block for Pain Management in Patients with Rib Fractures: A Systematic Review and Meta-Analysis of Randomized Controlled Trials

**DOI:** 10.3390/medicina62020281

**Published:** 2026-01-29

**Authors:** Abdullah M. Alharran, Sara Almutawtah, Sarah Saqer Alblooshi, Fahad A. Alsaid, Mohammad Salem Alajmi, Muneera Jasim AlRumaihi, Sara Ahmed Albuhmaid

**Affiliations:** 1 College of Medicine and Medical Sciences, Arabian Gulf University, Manama P.O. Box 26671, Bahrain; fahad.abdulnasser1@gmail.com (F.A.A.); muneerajjr@gmail.com (M.J.A.); saraamb@agu.edu.bh (S.A.A.); 2 Kuwait Institute for Medical Specialization, Kuwait City P.O. Box 1793, Kuwait; saraalmutawtah1@gmail.com; 3 Department of Surgery, Al Jahra Hospital, Kuwait City 13001, Kuwait; alblooshisarah66@gmail.com; 4 Faculty of Medicine and Health Sciences, Jordan University of Science and Technology, Amman 22110, Jordan; mohammadaldlaim@gmail.com

**Keywords:** SAPB, pain management, analgesia, anesthesia, chest trauma

## Abstract

*Background and Objectives*: Rib fractures cause intense pain, leading to respiratory complications. Standard care relies on systemic opioids, which carry significant adverse effects. The serratus anterior plane block (SAPB) has emerged as a promising regional anesthetic technique, but its efficacy remains unclear. This systematic review and meta-analysis of randomized controlled trials (RCTs) aimed to evaluate the efficacy and safety of SAPB versus standard care in patients with rib fractures. *Materials and Methods*: A comprehensive search of PubMed, Scopus, CENTRAL, and Web of Science was conducted for RCTs comparing SAPB to standard care in adults with rib fractures. The primary outcome was the pain score. Secondary outcomes included 24 h opioid consumption, need for rescue analgesia, and complications. Standardized mean differences (SMD) and risk ratios (RR) were pooled, using STATA SE 19.5. *Results*: Three RCTs involving 310 patients were included. SAPB significantly decreased pain scores at 2 h (SMD: −1.30, 95% CI [−2.39, −0.20]; *p* = 0.02), 6 h (SMD: −0.75, 95% CI [−1.41, −0.09]; *p* = 0.03), 12 h (SMD: −0.37, 95% CI [−0.68, −0.07]; *p* = 0.02), and 24 h (SMD: −5.67, 95% CI [−9.90, −1.43]; *p* = 0.01). This was associated with a significant reduction in 24 h opioid consumption (SMD: −0.45, 95% CI [−0.69, −0.21]; *p* < 0.001). However, no significant differences were found in the need for rescue analgesia (RR: 1.06, 95% CI [0.97, 1.16]; *p* = 0.18). *Conclusions*: SAPB provides significant short-term analgesic benefits and reduces opioid consumption in patients with acute rib fractures. While it appears safe, the current evidence is limited by a small number of trials and is insufficient to recommend SAPB as a first-line management option over standard care.

## 1. Introduction

Rib fractures are the most common injury following chest trauma [1]. The resulting pain from rib fractures is usually intense and difficult to manage [2]. This intense pain limits the chest wall’s normal movements, with symptoms like guarding, hypoventilation, difficulty coughing, and shallow breathing, which together increase the risk of lung collapse [3,4]. This sequence of poor pain control and respiratory dysfunction is directly linked to a high incidence of major complications [5], including retention of pulmonary secretions, nosocomial pneumonia, respiratory failure, and the need for mechanical ventilation [6]. Therefore, optimal pain management after rib fracture is critical.

The standard care for rib fracture pain remains patient-tailored multimodal pain management [7]. This strategy usually focuses on systemic analgesics, starting with routine paracetamol and non-steroidal anti-inflammatory drugs (NSAIDs) [8]. However, the primary treatment for severe pain depends on systemic opioids, administered as intravenous (IV) morphine infusions or via Patient-Controlled Analgesia (PCA) [8,9]. Still, opioids can cause several adverse events, including delirium, constipation, sedation, or ileus [10]. Also, opioids can cause respiratory depression, which can directly exacerbate the underlying pulmonary compromise in this patient population [11].

Thus, investigating other pain management options is necessary, such as local nerve block techniques. Recent trends in thoracic anesthesia have demonstrated a marked shift from neuraxial techniques, such as thoracic epidural analgesia, toward fascial plane blocks [7,12]. This transition is largely driven by the favorable safety profile of fascial blocks, which avoid the risks of hypotension and spinal hematoma associated with neuraxial techniques, making them valuable for trauma patients who may be coagulopathic or require anticoagulation [13].

In this regard, the serratus anterior plane block (SAPB) has emerged as a promising regional anesthesia technique. SAPB is an ultrasound-guided fascial plane block that provides targeted analgesia to the hemithorax [14]. This process requires injecting a local anesthetic into the fascial plane around the serratus anterior muscle, specifically targeting the lateral cutaneous branches of the thoracic intercostal nerves [15]. This technique offers a wide-ranging nerve block, usually affecting the T2 to T9 dermatomes, potentially decreasing the need for systemic opioids and their adverse effects [16]. This offers significant pain control while avoiding the adverse events associated with opioid analgesics.

Recently, some systematic reviews have attempted to synthesize the evidence for regional anesthesia in blunt thoracic trauma [7,17,18]. However, these reviews have either analyzed SAPB collectively with other fascial plane blocks, such as the erector spinae plane block [18], or utilized network meta-analysis that relies on indirect comparisons [7]. Also, earlier reviews were limited by the small sample sizes of available trials and the inclusion of observational data [17]. The recent publication of the large multicenter SABRE trial provides strong, novel, high-quality evidence that warrants a dedicated synthesis [19]. Therefore, we conducted this systematic review and meta-analysis to provide a focused and up-to-date synthesis specifically of RCTs comparing SAPB versus standard care in patients with rib fractures.

## 2. Methodology

### 2.1. Protocol Registration

This systematic review was registered in PROSPERO [CRD420251183783]. The methods for this systematic review and meta-analysis complied with the Preferred Reporting Items for Systematic Reviews and Meta-Analyses (PRISMA) guidelines [20] and the Cochrane Handbook for Systematic Reviews of Interventions [21].

### 2.2. Data Sources and Search Strategy

A systematic literature search was performed on 30 October 2025 by [A.M.A, S.A] across the following electronic databases: PubMed, Scopus, CENTRAL, and Web of Science. The search strategy utilized a combination of keywords and MeSH terms, including (“rib fracture*” OR “flail chest” OR “blunt chest trauma” OR “thoracic trauma” OR “chest wall injury”) AND (“serratus anterior plane block” OR “serratus plane block” OR “serratus block” OR “SAPB”). A complete overview of search terms and database results is presented in Table S1. Also, we manually reviewed the reference sections of all eligible trials to ensure complete coverage and avoid excluding any relevant studies.

### 2.3. Eligibility Criteria

Randomized Controlled Trials (RCTs) were included if they followed the following Population, Intervention, Control, and Outcome (PICO) criteria:

- Population (P): Adult patients with acute pain from traumatic rib fractures.
- Intervention (I): SAPB, regardless of anesthetic, volume, or technique (e.g., superficial or deep plane).
- Control (C): Standard of care, which included multimodal oral/IV analgesia or patient-controlled analgesia (PCA) with opioids.
- Outcomes (O): The primary outcome was the pain score at different time points post-intervention. Secondary outcomes included total opioid consumption, need for rescue analgesia, LoS, and complications.

### 2.4. Study Selection

Utilizing Covidence, the eligibility of the retrieved records was independently assessed by two reviewers (A.M.A, S.S.A). Following the automated removal of duplicates by Covidence, the remaining articles underwent screening in two phases. Initially, titles and abstracts underwent screening, followed by an evaluation of the full text of potentially eligible studies. Discrepancies among reviewers were resolved through discussion.

### 2.5. Data Extraction

Data extraction was independently performed by two reviewers (F.A.A, M.S.A). Any inconsistencies were resolved through discussion and consultation with the senior author. The data extraction process involved creating an Excel spreadsheet, which underwent pilot testing before formal extraction. The extraction form was organized into three main categories:

- Study characteristics: Study ID, country, study design, total number of patients, SAPB details, control group details, main inclusion criteria, primary outcome, and follow-up duration.
- Participant baseline characteristics: Age, gender, ASA status, BMI, number of rib fractures, and location of rib fractures.
- Outcome data: Pain scores at all reported time points, total opioid/analgesic consumption, LoS, need for further analgesia, and complications.

Dichotomous data were extracted as the number of events and total participants, whereas continuous data were extracted as the mean and standard deviation. We utilized the formulas proposed by Wan et al. [22] to convert data reported as median and interquartile range into mean and standard deviation.

### 2.6. Risk of Bias Assessment and Certainty of Evidence Evaluation

Methodological quality was evaluated for each RCT using the revised Cochrane Collaboration’s Risk of Bias tool (RoB 2) [23]. Two reviewers (M.J.A, S.A.A) independently assessed each study across the five domains (randomization process, deviations from intended interventions, missing outcome data, measurement of the outcome, and selection of the reported result). Disagreements were resolved by discussion among the reviewers to reach a consensus. Additionally, the overall certainty of the evidence was assessed using the Grading of Recommendations Assessment, Development, and Evaluation (GRADE) approach [24,25], which considers risk of bias, inconsistency, indirectness, imprecision, and publication bias. Each factor was carefully considered, and the reasoning behind every decision was clearly explained, resolving differences through open discussion.

### 2.7. Statistical Analysis

The statistical analyses were performed using Stata/SE version 19.5 (StataCorp., College Station, TX, USA). For continuous outcomes, the standardized mean difference (SMD) was calculated for pain and total opioid consumption, as studies used different assessment tools or units, respectively. To interpret the magnitude of SMD, we applied Cohen’s criteria: an SMD of approximately 0.2 was considered a small effect, 0.5 a moderate effect, and 0.8 or greater a large effect [26]. The Risk Ratio (RR) was calculated for dichotomous outcomes. The default analysis model was a fixed-effect model; however, a random-effects model was used if significant heterogeneity was detected. Heterogeneity was evaluated using the chi-squared (χ^2^) test and the I^2^ statistic. A *p*-value less than 0.1 for the χ^2^ test or an I^2^ value of 50% or higher indicated significant heterogeneity. In case of significant heterogeneity, a leave-one-out sensitivity analysis was performed to investigate the stability of the results. Finally, an assessment of publication bias was not possible, as all analyzed outcomes included fewer than 10 RCTs [27].

## 3. Results

### 3.1. Search Results and Study Selection

**Figure 1:**
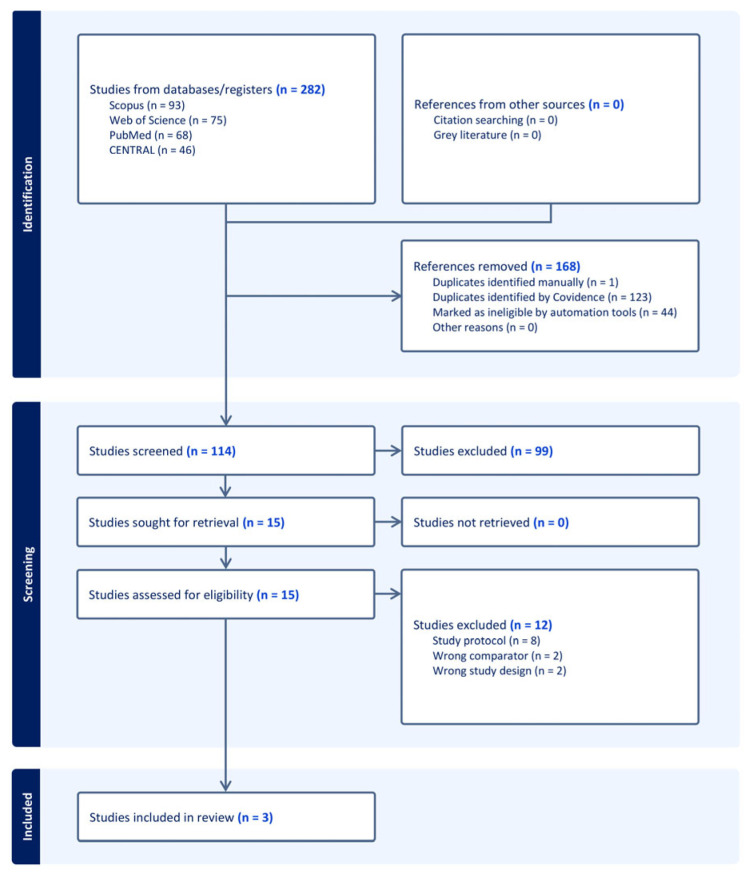
PRISMA flow chart of the screening process.

A total of 282 records were identified through database searching. After removing 168 irrelevant records, 114 records remained for screening. During the screening process, 99 records were excluded. Then, 15 full texts were assessed for eligibility. Of these, 12 studies were excluded for different reasons (Table S2). Finally, three trials [5,19,28] met the inclusion criteria and were included in the systematic review (Figure 1).

### 3.2. Characteristics of Included Studies

This review included three RCTs and 310 patients [5,19,28]. The studies were conducted across three different countries: Egypt, Australia, and Turkey. SAPB intervention consisted of a single-shot, ultrasound-guided block, though a specific anesthetic regimen that differed between the trials. Further details on the study design of the included trials are outlined in Table 1. Also, details on the included patients’ baseline data are outlined in Table 2.

### 3.3. Risk of Bias and Certainty of Evidence

**Figure 2:**
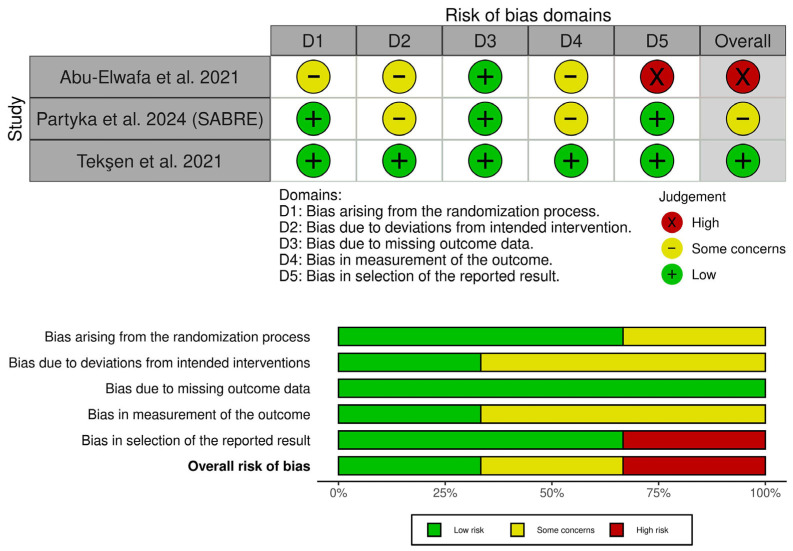
Quality assessment of risk of bias in the included trials. The upper panel presents a schematic representation of risks (low = green, unclear = yellow, and high = red) for specific types of biases of the studies in the review. The lower panel presents risks (low = red, unclear = yellow, and high = red) for the subtypes of biases of the combination of studies included in this review [5,19,28].

Tekşen et al. showed an overall low risk of bias [28]. Partyka et al. raised concerns about bias due to the open-label interventions and assessment of subjective outcomes [19]. Abu-Elwafa et al. showed a high risk of bias due to several problems, including selection bias (lack of randomization details), performance bias (open-label interventions), detection bias (open-label assessment of subjective outcomes), and reporting bias (lack of a registered protocol or ethical review board ID, with absent outcomes that were reported as assessed in the methods) [5] (Figure 2). Additionally, the certainty of evidence assessment details is reported in Table 3.

### 3.4. Primary Outcome: Pain Score

**Figure 3:**
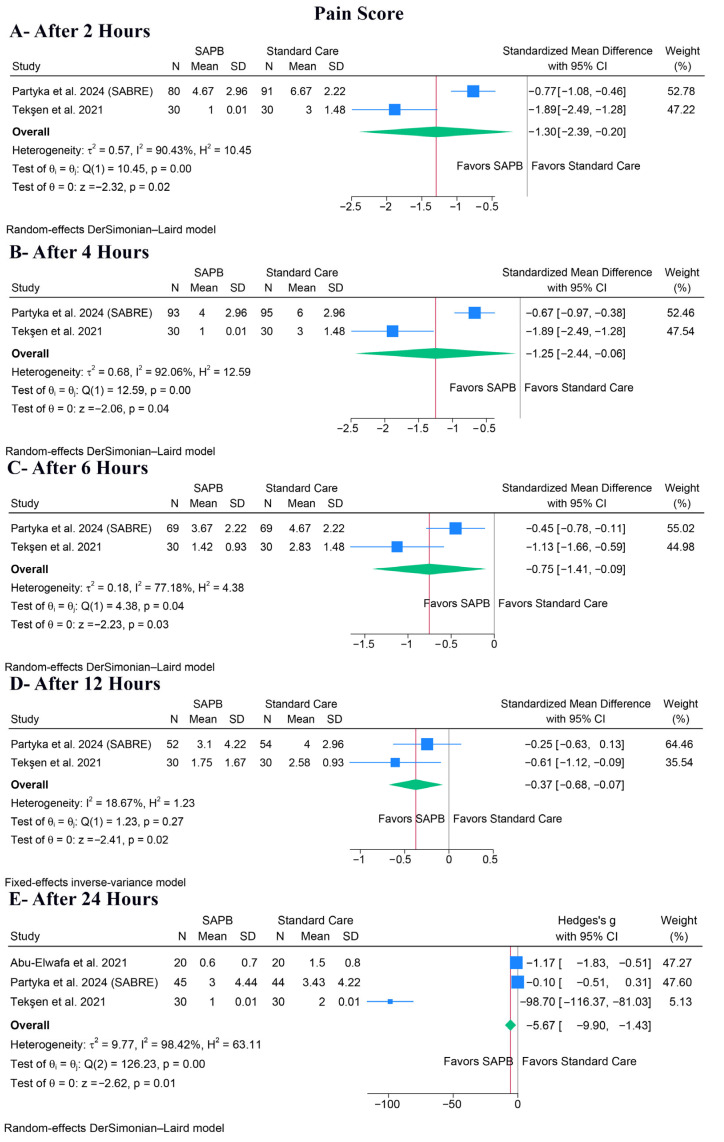
Forest plots of the primary outcome (pain score), CI: confidence interval [5,19,28].

SAPB significantly decreased pain scores at all measured time points, including after 2 h (SMD: −1.30, 95% CI [−2.39, −0.20], *p* = 0.02, I^2^ = 90.43%) (Figure 3A), after 4 h (SMD: −1.25, 95% CI [−2.44, −0.06], *p* = 0.04, I^2^ = 92.06%) (Figure 3B), after 6 h (SMD: −0.75, 95% CI [−1.41, −0.09], *p* = 0.03, I^2^ = 77.18%) (Figure 3C), after 12 h (SMD: −0.37, 95% CI [−0.68, −0.07], *p* = 0.02, I^2^ = 18.67%) (Figure 3D), and after 24 h (SMD: −5.67, 95% CI [−9.90, −1.43], *p* = 0.01, I^2^ = 98.42%) (Figure 3E).

For the pain score after 24 h, which exhibited extreme heterogeneity (I^2^ = 98.42%), a leave-one-out sensitivity analysis showed that the result became statistically non-significant after omitting any of the included studies (Figure S1). Also, the Galbraith plot suggested that Tekşen et al. was a potential source of the observed heterogeneity (Figure S2). Despite heterogeneity being noted in several other timepoints, we could not investigate the heterogeneity further, as only two trials reported it.

### 3.5. Secondary Outcomes

#### 3.5.1. Analgesia Consumption

**Figure 4:**
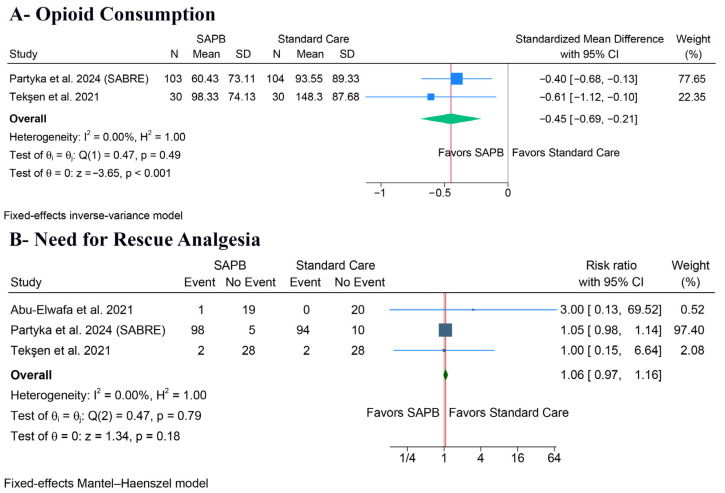
Forest plots of the secondary outcomes (analgesia consumption), CI: confidence interval [5,19,28].

Regarding 24 h opioid consumption, SAPB was associated with a significant decrease (SMD: −0.45, 95% CI [−0.69, −0.21], *p* < 0.001, I^2^ = 0%) (Figure 4A). However, there was no significant difference between the groups regarding the need for rescue analgesia (RR: 1.06, 95% CI [0.97, 1.16], *p* = 0.18, I^2^ = 0%) (Figure 4B).

#### 3.5.2. Health Services and Long-Term Outcomes

Regarding the length of stay, Partyka et al. reported no significant difference in either ICU LoS (Median 2.4 days for SAPB vs. 1.8 days for control, *p* = 0.91 $) or hospital LoS (Median 4.2 days for SAPB vs. 5 days for control, *p* = 0.3) [19]. Abu-Elwafa et al. listed ICU LoS as an outcome, but no results were reported [5]. Additionally, 30-day mortality was reported by Partyka et al. and was not significantly different between groups (1.2% for SAPB vs. 3.5% for control, *p* = 0.62) [19]. Finally, Partyka et al. measured 30-day quality of life using the EQ-5D-5L and found no measurable differences between the groups [19].

#### 3.5.3. Complications

The respiratory complications were assessed by Partyka et al., who found no significant difference in the rates of pneumonia (10% in the SAPB group vs. 11% in the control group). In that same trial, two patients in the SAPB group required mechanical ventilation compared to none in the control group, while one patient in each group required non-invasive ventilation [19]. Also, Tekşen et al. measured peripheral oxygen saturation (SpO_2_) and reported that it was significantly higher in the SAPB group at both 1 h (*p* = 0.012) and 24 h (*p* = 0.008) compared to the control group [28].

Moreover, delirium was measured by Partyka et al., who found a higher incidence (though not statistically significant, *p* = 0.09) in the SAPB group (20%) compared to the control group (9%) [19]. Regarding opioid-related side effects, Tekşen et al. reported nausea and vomiting in three control group patients (due to tramadol) and none in the SAPB group [28]. Similarly, Abu-Elwafa et al. reported two cases of constipation in the control (IV morphine) group and none in the SAPB group [5].

## 4. Discussion

This review, synthesizing data from three RCTs and 310 patients, found that SAPB provides statistically superior analgesia compared to standard care for rib fracture pain. This benefit was observed at all pooled time points within the first 24 h (2, 4, 6, 12, and 24 h). A significant reduction in 24 h opioid consumption integrated this analgesic advantage. However, these analgesic benefits did not translate into a significant decrease in the need for rescue analgesia, nor did they impact outcomes like ICU or hospital length of stay.

The SAPB’s effectiveness is due to its specific anatomical target. The block specifically targets the lateral cutaneous branches of the thoracic intercostal nerves, which are responsible for sensation in the anterolateral and posterior thorax [16]. The anesthetic blocks these nerves across a wide dermatomal range, from T2 to T9 [14,16]. This extensive block explains its utility for rib fractures, which often span multiple thoracic levels. Along with the intercostal nerves, the block can extend to the intercostobrachial, long thoracic, and thoracodorsal nerves, which further contribute to analgesia of the hemithorax [28].

Two unique techniques are described for performing SAPB: a superficial and a deep injection. The included studies used the superficial SAPB, involving the injection of local anesthetic into the plane superficial to the serratus anterior muscle (between the serratus anterior and latissimus dorsi muscles) [5]. This approach is often considered easier to perform and may achieve a wider spread of anesthetic [14]. Alternatively, a deep SAPB administers the anesthetic deep to the serratus anterior muscle (between the serratus anterior and the external intercostal muscles) [29]. Although deep injection facilitates anesthetic dispersion through respiratory movements [30], the superficial approach blocks the long thoracic nerve more effectively. It is, however, crucial to acknowledge that the SAPB does not impact the posterior rami or the anterior cutaneous branches of the intercostal nerves, thereby not replicating the comprehensive hemithoracic anesthesia achieved by methods such as thoracic epidural anesthesia [30].

It is also notable that the included trials utilized single-shot techniques. However, the use of a continuous SAP catheter may provide superior and more sustained pain control compared to a single injection. This can further avoid the need for rescue analgesics and decrease the length of hospital stay [31,32]. Also, the clinical efficacy of the block likely depends on the complexity of the trauma. To clarify, factors such as the presence of bilateral rib fractures, flail chest, and a higher total number of fractured ribs generally mandate more aggressive multimodal analgesia that a unilateral single-shot block may not fully address [33,34].

The primary finding of better analgesia is most marked in the immediate acute phase. The pooled data indicate the strongest effect size in the first 2–6 h, which is consistent with the expected pharmacokinetics of a single-shot regional block using long-acting local anesthetics [35]. The pooled effect at 24 h; however, showed extreme heterogeneity. Also, this unusually large effect size (SMD: −5.67) appears to be driven by Tekşen et al. [26], where the reported median pain scores exhibited very narrow interquartile ranges. The conversion of this non-parametric data to means and standard deviations resulted in extremely small variance estimates, which mathematically inflated the SMD [22]. Consequently, this specific point estimate should be interpreted with caution, as evidenced by the sensitivity analysis where the removal of Tekşen et al. [26] significantly changed the results.

Beyond statistical conversions, the observed heterogeneity likely reflects the significant clinical and methodological diversity among the included trials. Clinically, the control arm interventions varied: while Partyka et al. [19] employed a pragmatic standard care bundle that allowed for diverse analgesic regimens including oral opioids, Abu-Elwafa et al. [5] utilized a strict IV morphine protocol, and Tekşen et al. [26] used a tramadol PCA regimen. Such variations in the standard of care may have led to different baseline pain levels. Also, methodologically, differences in pain scales (VAS vs. NRS) and the timing of assessments related to the block performance further contribute to the observed heterogeneity. Despite these differences, the direction of the effect remained consistent across studies, favoring SAPB.

Additionally, a significant clinical advantage of regional anesthesia is its opioid-sparing effect. This significant analgesic benefit was reflected in the substantial reduction in opioid consumption up to 24 h. The data strongly suggest that SAPB is an effective method for reducing patients’ reliance on systemic opioids. However, the reduction in pain and opioid use was not consistent with the lack of difference in the need for rescue analgesia. The heterogeneous “rescue analgesia” definition in each trial is the key to understanding this contradiction. Partyka et al. reported that the “rescue analgesia” was not for ineffective analgesia [19]. They noted that approximately 27–28% of both groups were given further regional blocks as a standard escalation in care when the initial block’s effects subsided [19]. In contrast, the control groups in the other trials were already being treated with strong painkillers, such as IV morphine infusion and tramadol PCA [5,28].

Moreover, SAPB appears to be a safe procedure. All trials, encompassing 153 patients receiving the block, reported zero block-related complications such as local anesthetic systemic toxicity, pneumothorax, or hematoma [5,19,28]. Specifically, no patients experienced pneumothorax, vascular injury, hematoma, or local anesthetic systemic toxicity. This is consistent with the established safety record of ultrasound-guided fascial plane blocks [36]. Also, the opioid-sparing impact yielded obvious benefits; the SAPB groups reported fewer opioid-related adverse effects, such as nausea/vomiting and constipation. Still, Partyka et al. observed a non-significant trend toward increased delirium within the SAPB cohort [19]. This requires careful consideration, despite the authors’ recognition that their SAPB group exhibited a higher baseline comorbidity burden [19]. Finally, Tekşen et al. reported that SAPB significantly reduced the incidence of chronic pain at three months [28]. Given the prevalence of chronic pain after thoracic trauma, which can affect up to 50% of patients [37,38], this observation supports the potential for a long-term, preemptive analgesic benefit that warrants further study.

Furthermore, contextualizing SAPB within the broader context of regional anesthesia is essential. While thoracic epidural analgesia and paravertebral block have historically been considered the gold standards, their application is often limited by risks of hypotension and spinal hematoma, particularly in coagulopathic trauma patients [7]. In contrast, SAPB is a superficial block with a superior safety profile. Recently, the erector spinae plane (ESP) block has emerged as another regional option that provides analgesia for posterior and lateral rib fractures [18]. However, a distinct advantage of SAPB is its feasibility in the supine position. Unlike the ESP block, which typically requires patient repositioning, SAPB is technically less challenging to perform in immobilized trauma patients while showing a comparable opioid-sparing efficacy [7,13].

## 5. Clinical Implications

Accordingly, while SAPB may not yet totally replace the current standard neuraxial techniques, it should be prioritized in specific clinical scenarios. Notably, SAPB can be the preferred regional option for patients with contraindications to neuraxial anesthesia, such as coagulopathy or therapeutic anticoagulation, as it avoids the risk of spinal hematoma associated with epidural or paravertebral blocks [13]. It is also perfectly suited for the acute trauma setting, as it can be performed rapidly in the emergency department on supine patients maintained on spinal precautions [15,19]. Finally, for elderly patients or those with opioid intolerance, SAPB serves as a critical opioid-sparing adjunct to mitigate delirium and respiratory depression [12].

## 6. Limitations

The primary limitation is the small number of included studies, which restricts the power of all pooled analyses. Second, two of the three trials had multiple concerns of bias. Third, the control groups received different regimens of standard of care, from standard care bundles to IV morphine and tramadol PCA. Fourth, the pain score analyses suffered from high statistical heterogeneity, making the magnitude of the pooled effect uncertain. Fifth, there was some variability in the SAPB technique; while predominantly superficial, the specific local anesthetic regimens and volumes varied across included studies, which may have influenced the duration and efficacy of the block. Sixth, this review could not pool functional respiratory outcomes, as they were not reported in the included trials. Seventh, long-term follow-up data remain limited, with only one trial assessing the incidence of chronic pain at three months [28], restricting our ability to draw conclusions regarding the prevention of chronic post-traumatic pain. Finally, these limitations affected the GRADE certainty of evidence assessment, rendering it either low or very low.

## 7. Conclusions

SAPB provides significant analgesic benefits and reduces opioid consumption in patients with acute rib fractures. While it showed an excellent safety profile, the current evidence, based on a small number of trials, is insufficient to consider SAPB as a first-line management option for rib fractures. Accordingly, future research should prioritize large-scale, multicenter RCTs to establish definitive practice guidelines. These future trials should aim to standardize the SAPB technique and employ consistent multimodal control regimens to minimize the clinical heterogeneity observed in current evidence.

## Figures and Tables

**Table 1:** Summary characteristics of the included RCTs.

Study ID	Study Design	Country	Total Participants	SAPB Details	Control Details	Adjuvant Analgesia	Primary Outcome	Pain Assessment Score	Main Inclusion Criteria	Follow-Up Duration
Abu-Elwafa et al. 2021 [5]	RCT	Egypt	40	Technique: Ultrasound-guided (Superficial). Regimen: Bupivacaine 0.25% (15 mL) + Lidocaine 1% (15 mL). Total Volume: 30 mL	Drug: IV Morphine. Dosing: 0.1 mg/kg loading dose, 10–20 ug/kg/h infusion. Administration: Intravenous infusion.	Rescue analgesia (paracetamol, ketorolac) was available for both groups.	Pain score	VAS (0–10)	Patients >15 years. Lateral/anterior multiple rib fractures (3–6 ribs). Excruciating pain is not responding to conventional analgesics.	24 h
Partyka et al. 2024 (SABRE) [19]	RCT	Australia	210	Technique: Ultrasound-guided (Superficial or Deep). Regimen: Ropivacaine 0.375%. Total Volume: 40 mL	Standard Care. Included protocolized rib fracture care bundles with multimodal oral or intravenous analgesia.	Both groups received protocolized rib fracture care bundles, including multimodal analgesia (e.g., paracetamol, NSAIDs).	Composite pain score at 4 h (defined as pain score reduction ≥ 2 points AND absolute pain score < 4).	NRS (0–10)	Patients ≥16 years. Clinically suspected or radiologically proven rib fractures.	30 days
Tekşen et al. 2021 [28]	RCT	Turkey	60	Technique: Ultrasound-guided (Superficial). Regimen: Bupivacaine 0.25%. Total Volume: 30 mL	The control group received PCA without a block. Comparator Details: Drug: IV Tramadol. Dosing: 10 mg per press. Administration: PCA	All patients in both groups received IV tramadol PCA.	Total tramadol consumption in 24 h.	NRS (0–10)	Patients aged 18–90 years. Rib fracture pain. Baseline NRS pain score ≥ 4.	24 h

RCT: Randomized Controlled Trial. SAPB: Serratus Anterior Plane Block. IV: Intravenous. ug/kg/h: Micrograms per kilogram per hour. mg/kg: Milligrams per kilogram. mL: Milliliter. NSAIDS: Nonsteroidal Anti-inflammatory Drugs. PCA: Patient-Controlled Analgesia. VAS: Visual Analog Scale. NRS: Numerical Rating Scale. N: Number.

**Table 2:** Baseline characteristics of the participants.

Study ID	Number of Participants in Each Group		Age (Years), Mean (SD)		Gender (Male/Female)		ASA I/II/III/IV		Rib Fracture Location		Number of Rib Fractures	
	SAPB	Standard Care	SAPB	Standard Care	SAPB	Standard Care	SAPB	Standard Care	SAPB	Standard Care	SAPB	Standard Care
Abu-Elwafa et al. 2021 [5]	20	20	NR	NR	NR	NR	NR	NR	Lateral or anterior (Inclusion criteria)	Lateral or anterior (Inclusion criteria)	3 to 6 ribs (Inclusion criteria)	3 to 6 ribs (Inclusion criteria)
Partyka et al. 2024 (SABRE) [19]	103	104	71 (54–85)	71 (58–82)	67/36	62/42	NR	NR	A: 8, L: 58, P: 17	A: 9, L: 57, P: 17	4 (2–5)	3 (2–5)
Tekşen et al. 2021 [28]	30	30	50.7 (18.8)	42.4 (15.8)	11/19	7/23	14/13/3	7/22/1	P: 15, L: 5, A: 10	P: 11, L: 10, A: 9	NR	NR

N: Number. SAPB: Serratus Anterior Plane Block. SD: Standard Deviation. NR: Not Reported. ASA: American Society of Anesthesiologists. A: Anterior. L: Lateral. P: Posterior.

**Table 3:** GRADE evidence profile.

Certainty Assessment							Summary of Findings				
Participants (Studies) Follow-Up	Risk of Bias	Inconsistency	Indirectness	Imprecision	Publication Bias	Overall Certainty of Evidence	Study Event Rates (%)		Relative Effect (95% CI)	Anticipated Absolute Effects	
							with [SoC]	with [SAPB]		Risk with [SoC]	Risk Difference with [SAPB]
Pain Score at 2 h											
231 (2 RCTs)	serious _a_	very serious _b_	not serious	very serious _c,d_	none	⨁◯◯◯ Very low _a,b,c,d_	-	-	-	-	SMD 1.3 SD lower (2.39 lower to 0.2 lower)
Pain Score at 4 h											
248 (2 RCTs)	serious _a_	very serious _b_	not serious	very serious _c,d_	none	⨁◯◯◯ Very low _a,b,c,d_	-	-	-	-	SMD 1.25 SD lower (2.44 lower to 0.06 lower)
Pain Score at 6 h											
198 (2 RCTs)	serious _a_	very serious _b_	not serious	very serious _c,d_	none	⨁◯◯◯ Very low _a,b,c,d_	-	-	-	-	SMD 0.75 SD lower (1.41 lower to 0.09 lower)
Pain Score at 12 h											
166 (2 RCTs)	serious _a_	not serious	not serious	serious _d_	none	⨁⨁◯◯ Low _a,d_	-	-	-	-	SMD 0.37 SD lower (0.68 lower to 0.07 lower)
Pain Score at 24 h											
189 (3 RCTs)	serious _a_	very serious _b_	not serious	very serious _c,d_	none	⨁◯◯◯ Very low _a,b,c,d_	-	-	-	-	SMD 5.67 SD lower (9.9 lower to 1.43 lower)
Opioid Consumption (24 h)											
267 (2 RCTs)	serious _a_	not serious	not serious	very serious _c,d_	none	⨁◯◯◯ Very low _a,c,d_	-	-	-	-	SMD 0.45 SD lower (0.69 lower to 0.21 lower)
Need for Rescue Analgesia											
307 (3 RCTs)	serious _a_	not serious	not serious	serious _e_	none	⨁⨁◯◯ Low _a,e_	12/154 (7.8%)	8/153 (5.2%)	RR 1.06 (0.97 to 1.10)	12/154 (7.8%)	5 more per 1000 (from 2 fewer to 8 more)

CI: confidence interval; RR: risk ratio; SMD: standardized mean difference; Explanations: _a_. Partyka et al. raised concerns about bias due to the open-label interventions and assessment of subjective outcomes. Abu-Elwafa et al. [5] showed a high risk of bias due to several problems, including selection bias (lack of randomization details), performance bias (open-label interventions), detection bias (open-label assessment of subjective outcomes), and reporting bias (lack of a registered protocol or ethical review board ID, with absent outcomes that were reported as assessed in the methods). _b_. I^2^ > 75%. _c_. A wide confidence interval. _d_. Low number of participants. _e_. Low number of events.

## Data Availability

No new data were created or analyzed in this study.

## Supplementary Materials

The following supporting information can be downloaded at: https://www.mdpi.com/article/10.3390/medicina62020281/s1, Figure S1: Leave-one-out sensitivity analysis of pain score after 24 h; Figure S2: Galbraith plot of pain score after 24 h; Table S1: Search strategy; Table S2: Excluded records in full-text screening; Table S3: PRISMA 2020 Checklist.
