# Supplementary material for: Efficacy and Safety of Serratus Anterior Plane Block for Pain Management in Patients with Rib Fractures: A Systematic Review and Meta-Analysis of Randomized Controlled Trials
Source: Medicina (Kaunas). 2026 Jan 29;62(2):281. doi: 10.3390/medicina62020281 (PMC12943399; doi:10.3390/medicina62020281)
Supplement: Supplementary file 1 [file medicina-62-00281-s001.zip › medicina-3998402-supplementary.pdf]

Supplementary Materials:

Contents:

Tables.

Table S1: Search strategy.

Table S2: Excluded records in full-text screening.

Table S3: PRISMA 2020 Checklist.

Figures.

Figure S1: Leave-one-out sensitivity analysis of pain score after 24 hours.

Figure S2: Galbraith plot of pain score after 24 hours.

Table S1: Search Strategy.

**Database**	**Search Terms**	**Search Field**	**Search Results**
Pubmed	("rib fracture*" OR "flail chest" OR "blunt chest trauma" OR "thoracic trauma" OR "chest wall injury") AND ("serratus anterior plane block" OR "serratus plane block" OR "serratus block" OR "SAPB")	All Fields	68
Cochrane	("rib fracture*" OR "flail chest" OR "blunt chest trauma" OR "thoracic trauma" OR "chest wall injury") AND ("serratus anterior plane block" OR "serratus plane block" OR "serratus block" OR "SAPB")	All Text	46
Web of Science	("rib fracture*" OR "flail chest" OR "blunt chest trauma" OR "thoracic trauma" OR "chest wall injury") AND ("serratus anterior plane block" OR "serratus plane block" OR "serratus block" OR "SAPB")	All Fields	75
SCOPUS	TITLE-ABS-KEY (("rib fracture*" OR "flail chest" OR "blunt chest trauma" OR "thoracic trauma" OR "chest wall injury") AND ("serratus anterior plane block" OR "serratus plane block" OR "serratus block" OR "SAPB"))	Title, Abstract, Keywords	93

Table S2: Excluded records in full-text screening.

Title	DOI	Study ID	Exclusion Reason
Comparison of the analgesic efficacy of ultrasound-guided superficial serratus anterior plane block and intercostal nerve block for rib fractures: a randomized controlled trial.	10.1186/s12871-025-03000-6	Zengin 2025	Wrong comparato r
An ultrasound-guided serratus anterior plane block with continuous local anaesthetic infusion and epidural analgesia for rib fracture pain.	10.1111/aas.14355	LundÃ©n 2024	Wrong comparato r
Ultrasound-Guided Serratus Anterior Plane Block (SAPB) Improves Pain Control in Patients with Rib Fractures.	10.1002/jum.15953	Kring 2022	Wrong study design
Ultrasound-Guided Serratus Anterior Plane Block for Rib Fracture-Associated Pain Management in Emergency Department.	10.4103/JETS.JETS_155_19	Paul 2020	Wrong study design
US-guided SAPB for Rib Fractures in the ED	-	NCT03619785 2018	Study protocol
Ultrasound Guided Serratus Anterior Plane Block in ED Patients with Rib Fractures	-	NCT06299137 2024	Study protocol
Ultrasound Guided Serratus Anterior Plane Block for Rib Fractures	-	NCT05748366 2023	Study protocol
Analgesic Efficacy of Serratus Anterior Plane Block in The Rib Fractures Pain	-	NCT04293354 2020	Study protocol
Ultrasound-Guided Continuous Serratus Anterior Plane Block vs Dexmedetomidine Infusion in Patients with Rib Fractures	-	NCT05965895 2023	Study protocol
Evaluation of Serratus Plane Block on the Respiratory Pattern in Patients with Multiple Rib Fractures	-	NCT05105399 2021	Study protocol
Serratus Plane Block with Parenteral Opioid Analgesia Versus Patient Controlled Analgesia in Rib Fractures	-	NCT03919916 2019	Study protocol
Serratus Plane Block for Rib Fractures	-	NCT03664973 2018	Study protocol

Table S3: PRISMA 2020 Checklist.

Section and Topic	Item #	Checklist item	Location where item is reported
**TITLE**			
Title	1	Identify the report as a systematic review.	Title page
**ABSTRACT**			
Abstract	2	See the PRISMA 2020 for Abstracts checklist.	Abstract section
**INTRODUCTION**			
Rationale	3	Describe the rationale for the review in the context of existing knowledge.	Introduction, paragraphs 1–5
Objectives	4	Provide an explicit statement of the objective(s) or question(s) the review addresses.	End of Introduction
**METHODS**			
Eligibility criteria	5	Specify the inclusion and exclusion criteria for the review and how studies were grouped for the syntheses.	Section 2.3 Eligibility Criteria
Information sources	6	Specify all databases, registers, websites, organisations, reference lists and other sources searched or consulted to identify studies. Specify the date when each source was last searched or consulted.	Section 2.2 Data Sources and Search Strategy
Search strategy	7	Present the full search strategies for all databases, registers and websites, including any filters and limits used.	Table S1 (Supplementary Materials)
Selection process	8	Specify the methods used to decide whether a study met the inclusion criteria of the review, including how many reviewers screened each record and each report retrieved, whether they worked independently, and if applicable, details of automation tools used in the process.	Section 2.4 Study Selection
Data collection process	9	Specify the methods used to collect data from reports, including how many reviewers collected data from each report, whether they worked independently, any processes for obtaining or confirming data from study investigators, and if applicable, details of automation tools used in the process.	Section 2.5 Data Extraction
Data items	10a	List and define all outcomes for which data were sought. Specify whether all results that were compatible with each outcome domain in each study were sought (e.g. for all measures, time points, analyses), and if not, the methods used to decide which results to collect.	Section 2.3 + 2.5
	10b	List and define all other variables for which data were sought (e.g. participant and intervention characteristics, funding sources). Describe	Section 2.5

Section and Topic	Item #	Checklist item	Location where item is reported
		any assumptions made about any missing or unclear information.	
Study risk of bias assessment	11	Specify the methods used to assess risk of bias in the included studies, including details of the tool(s) used, how many reviewers assessed each study and whether they worked independently, and if applicable, details of automation tools used in the process.	Section 2.6 Risk of Bias and Certainty of Evidence
Effect measures	12	Specify for each outcome the effect measure(s) (e.g. risk ratio, mean difference) used in the synthesis or presentation of results.	Section 2.7 Statistical Analysis
Synthesis methods	13a	Describe the processes used to decide which studies were eligible for each synthesis (e.g. tabulating the study intervention characteristics and comparing against the planned groups for each synthesis (item #5)).	Section 2.3 + 3.1
	13b	Describe any methods required to prepare the data for presentation or synthesis, such as handling of missing summary statistics, or data conversions.	Section 2.5 + Wan et al. conversion
	13c	Describe any methods used to tabulate or visually display results of individual studies and syntheses.	Forest plots (Figures 3, 4), Tables 1–3
	13d	Describe any methods used to synthesize results and provide a rationale for the choice(s). If meta-analysis was performed, describe the model(s), method(s) to identify the presence and extent of statistical heterogeneity, and software package(s) used.	Section 2.7 Statistical Analysis
	13e	Describe any methods used to explore possible causes of heterogeneity among study results (e.g. subgroup analysis, meta-regression).	Section 3.4 (sensitivity analysis, Galbraith plot)
	13f	Describe any sensitivity analyses conducted to assess robustness of the synthesized results.	Section 3.4 + Figure S1
Reporting bias assessment	14	Describe any methods used to assess risk of bias due to missing results in a synthesis (arising from reporting biases).	Section 2.7 (publication bias not assessed: <10 studies)

Section and Topic	Item #	Checklist item	Location where item is reported
Certainty assessment	15	Describe any methods used to assess certainty (or confidence) in the body of evidence for an outcome.	Section 2.6 + Table 3 (GRADE)
**RESULTS**			
Study selection	16a	Describe the results of the search and selection process, from the number of records identified in the search to the number of studies included in the review, ideally using a flow diagram.	Section 3.1 + Figure 1 (PRISMA flowchart)
	16b	Cite studies that might appear to meet the inclusion criteria, but which were excluded, and explain why they were excluded.	Table S2
Study characteristics	17	Cite each included study and present its characteristics.	Section 3.2 + Tables 1 & 2
Risk of bias in studies	18	Present assessments of risk of bias for each included study.	Section 3.3 + Figure 2
Results of individual studies	19	For all outcomes, present, for each study: (a) summary statistics for each group (where appropriate) and (b) an effect estimate and its precision (e.g. confidence/credible interval), ideally using structured tables or plots.	Tables 1–2, Figures 3–4
Results of syntheses	20a	For each synthesis, briefly summarise the characteristics and risk of bias among contributing studies.	Section 3.3
	20b	Present results of all statistical syntheses conducted. If meta-analysis was done, present for each the summary estimate and its precision (e.g. confidence/credible interval) and measures of statistical heterogeneity. If comparing groups, describe the direction of the effect.	Sections 3.4 & 3.5
	20c	Present results of all investigations of possible causes of heterogeneity among study results.	Section 3.4
	20d	Present results of all sensitivity analyses conducted to assess the robustness of the synthesized results.	Section 3.4 + Figure S1
Reporting biases	21	Present assessments of risk of bias due to missing results (arising from reporting biases) for each synthesis assessed.	Section 2.7
Certainty of evidence	22	Present assessments of certainty (or confidence) in the body of evidence for each outcome assessed.	Section 3.3 + Table 3
**DISCUSSION**			

Section and Topic	Item #	Checklist item	Location where item is reported
Discussion	23a	Provide a general interpretation of the results in the context of other evidence.	Section 4 Discussion
	23b	Discuss any limitations of the evidence included in the review.	Section 6 Limitations
	23c	Discuss any limitations of the review processes used.	Section 6 Limitations
	23d	Discuss implications of the results for practice, policy, and future research.	Section 5 Clinical Implications
**OTHER INFORMATION**			
Registration and protocol	24a	Provide registration information for the review, including register name and registration number, or state that the review was not registered.	Section 2.1 Protocol Registration
	24b	Indicate where the review protocol can be accessed, or state that a protocol was not prepared.	PROSPERO registration
	24c	Describe and explain any amendments to information provided at registration or in the protocol.	Not applicable
Support	25	Describe sources of financial or non-financial support for the review, and the role of the funders or sponsors in the review.	Funding section
Competing interests	26	Declare any competing interests of review authors.	Conflicts of Interest
Availability of data, code and other materials	27	Report which of the following are publicly available and where they can be found: template data collection forms; data extracted from included studies; data used for all analyses; analytic code; any other materials used in the review.	Data Availability Statement

From: Page MJ, McKenzie JE, Bossuyt PM, Boutron I, Hoffmann TC, Mulrow CD, et al. The PRISMA 2020 statement: an updated guideline for reporting systematic reviews. BMJ 2021;372:n71. doi: 10.1136/bmj.n71.

This work is licensed under CC BY 4.0. To view a copy of this license, visit https://creativecommons.org/licenses/by/4.0/

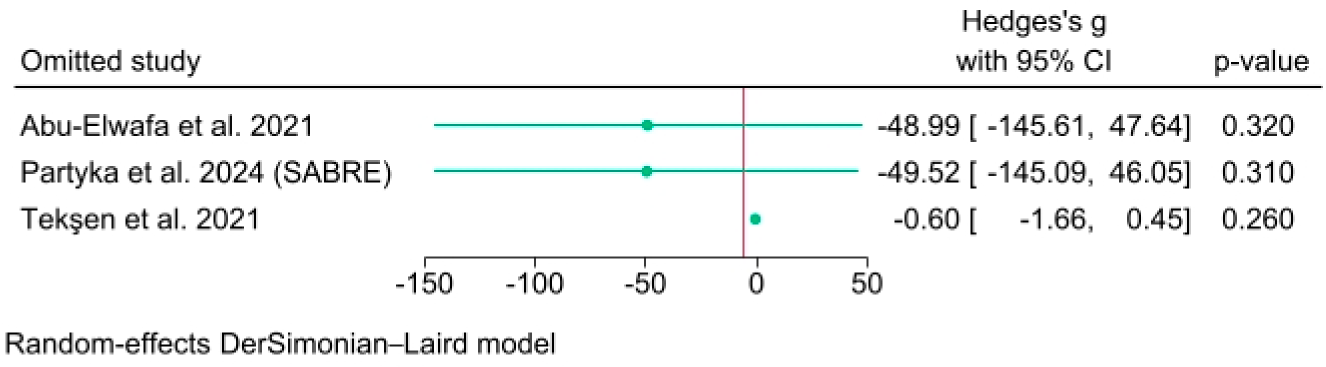

Figure S1: Leave-one-out sensitivity analysis of pain score after 24 hours.

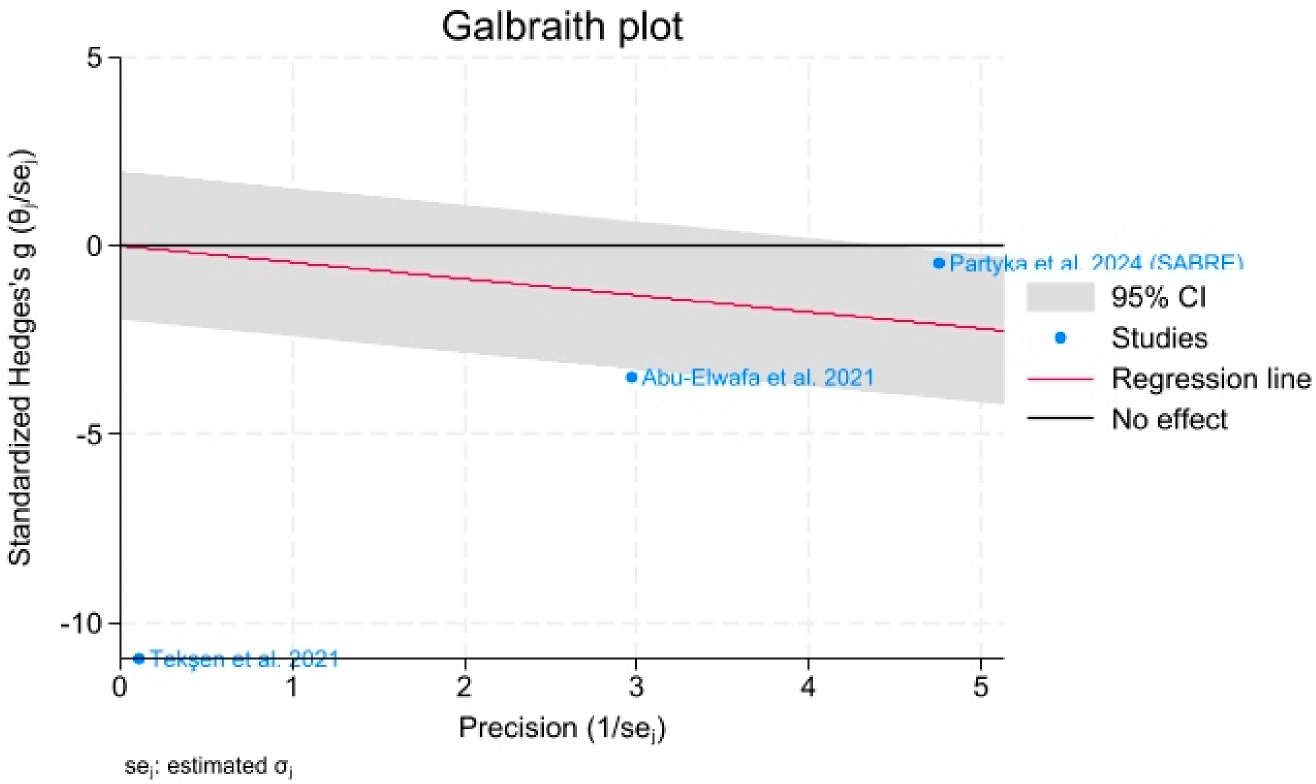

Figure S2: Galbraith plot of pain score after 24 hours.
